# Immune Checkpoint Inhibitor-Mediated Cancer Theranostics with Radiolabeled Anti-Granzyme B Peptide

**DOI:** 10.3390/pharmaceutics14071460

**Published:** 2022-07-13

**Authors:** Carolina de Aguiar Ferreira, Pedram Heidari, Bahar Ataeinia, Nicoleta Sinevici, Alyssa Granito, Hritik Mahajan Kumar, Eric Wehrenberg-Klee, Umar Mahmood

**Affiliations:** Department of Radiology, Massachusetts General Hospital, Boston, MA 02114, USA; cferreira@wisc.edu (C.d.A.F.); heidari.pedram@mgh.harvard.edu (P.H.); bataeinia@mgh.harvard.edu (B.A.); sinevicn@tcd.ie (N.S.); granito.a@northeastern.edu (A.G.); kumar.hr@northeastern.edu (H.M.K.); ewehrenberg-klee@partners.org (E.W.-K.)

**Keywords:** theranostics, immune checkpoint inhibitors, targeted radionuclide therapy

## Abstract

Although immune checkpoint inhibitors (ICI) have revolutionized cancer management, patient response can be heterogeneous, and the development of ICI resistance is increasingly reported. Novel treatment strategies are necessary not only to expand the use of ICI to previously unresponsive tumor types but also to overcome resistance. Targeted radionuclide therapy may synergize well with ICIs since it can promote a pro-inflammatory tumor microenvironment. We investigated the use of a granzyme B targeted peptide (GZP) as a cancer theranostic agent, radiolabeled with ^68^Ga (^68^Ga-GZP) as a PET imaging agent and radiolabeled with ^90^Y (^90^Y-GZP) as a targeted radionuclide therapy agent for combinational therapy with ICI in murine models of colon cancer. Our results demonstrate that GZP increasingly accumulates in tumor tissue after ICI and that the combination of ICI with ^90^Y-GZP promotes a dose-dependent response, achieving curative response in some settings and increased overall survival.

## 1. Introduction

Immune checkpoint inhibitors (ICI) have revolutionized cancer treatment. While conventional therapies rely on cytotoxic effects directly affecting tumor cells, ICIs work primarily at restoring antitumor immune responses by suppressing co-inhibitory T-cell signaling [1]. Even though the role of immune checkpoints is constantly evolving, ICI efficacy depends on the activation and expansion of CD8 T cells present in the tumor microenvironment (TME). For that, tumor antigens are recognized by T cells through antigen-presenting cells (APCs) via major histocompatibility complex (MHC) interaction. Activation is successful with an appropriate cytokine environment and a secondary signal (via CD28 receptor on T cells). The newly differentiated tumor-specific effector T cells proliferate and migrate to the TME where they release granzyme B and perforin to promote tumor cell death [2]. Effector memory T cells are also generated and can allow life-long immunologic memory [3,4]. Several co-receptors can be present at different molecular checkpoints. For example, CTLA-4 competes with CD28 and is involved in Regulatory T cells (Treg) suppressive function, while PD-L1 signal promotes T-cell exhaustion and immune suppression [1,5]. Anti-CLA-4 and anti PD1/PD-L1 monoclonal antibody therapies increase cytotoxic T-cell activation and have been approved and successfully employed for a diverse range of cancer types, including microsatellite instability-high colon adenocarcinoma [6,7,8]. Although ICIs have generated incredible results, patient response is variable and heterogeneous even among the same cancer type, while some patients do not respond at all. In addition, the development of ICI resistance is increasingly reported [9,10]. ICI failure and acquired resistance mechanisms can be due to the tumor, TME or patient immune system characteristics. In fact, more than one mechanism of resistance may be present within one tumor and patients with the same tumor type can have different failure mechanisms [11]. In this context, novel combination treatments able to expand ICI use or overcome ICI resistance are needed. Targeted radionuclide therapy (TRT) is a radiotherapy modality consisting of systemic delivery of radioactive atoms to induce DNA damage in tumor cells, with localized accumulation through targeting ligands. Consequently, TRT can deliver its tumoricidal radiation irrespective of tumor location and burden in a selective manner that minimizes collateral tissue damage [12]. TRT agents typically target specific receptors and metabolic transporters that are differentially overexpressed in tumor cells. Our group has previously developed a peptide-based radiotracer (^68^Ga-GZP) that can selectively bind to granzyme B released by activated CD8 T cells in response to ICI [13,14]. We have shown that granzyme B PET imaging with ^68^Ga-GZP can be used as a biomarker for predicting tumoral immunotherapy response [14] and can non-invasively identify immune-related adverse events [15]. Since GZP increasingly accumulates in the tumor tissue after immunotherapy, we investigated the use of GZP as a cancer theranostic agent, radiolabeled with ^68^Ga (^68^Ga-GZP) as a PET imaging agent and radiolabeled with ^90^Y (^90^Y-GZP) as a TRT agent for combinational therapy with ICI in murine models of colon cancer.

## 2. Materials and Methods

### 2.1. Syngeneic Colon Cancer Animal Model

Murine colon adenocarcinoma MC38 cells derived from C57BL6 mice (Kerafast, Boston, MA, USA) and murine colon carcinoma CT26 derived from BALB/c (ATCC) were cultured in DMEM or RPMI medium (respectively) supplemented with 10% FBS at 37 °C and 5% CO_2_.

All experimental procedures and animal studies were performed under the approval of the Institutional Animal Care and Use Committee (IACUC). Syngeneic allograft tumors were implanted subcutaneously in the upper right flank of mice (1 × 10^6^ cells in a 1:1 ratio in Matrigel).

### 2.2. Ga-68 and Y-90 Radiolabeling of GZP

Radiolabeling of NOTA-GZP was performed as previously described [14]. In brief, 100 µg (approximately 1e-7mols) of NOTA-GZP (in 100 µL of PBS) was mixed with 370 MBq of ^68^Ga in 2 M HEPES buffer (pH 3.5–4.0) for 10 min at room temperature. The reaction product was purified with a C18 Sep-Pak solid-phase extraction cartridge, eluted with 200 µL of 70% ethanol and diluted with saline to a final concentration of less than 10% ethanol prior to administration. Radiolabeling yield was calculated through instant thin-layer chromatography (iTLC) using two solvent systems as reported elsewhere [16,17].

^90^YCl_3_ was purchased from Eckert and Ziegler (Germany); 370 MBq of activity in 500 µL of sodium acetate buffer (pH 5.5) was mixed with 100 µg of DOTA-GZP (in 100 µL of PBS) for 1 h at 90 °C. The reaction was purified as described above and radiolabeling yield was calculated through iTLC using 50 mM EDTA as solvent.

### 2.3. PET/MR Imaging

Mice were intravenously injected with 7.4–11.11 MBq of ^68^Ga-GZ. PET/MR Images were acquired 1 h post injection (p.i) on anesthetized mice on a preclinical multimodal 4.7T MR/PET scanner (Bruker, Billerica, MA, USA). Static PET images were acquired for 15 min followed by acquisition of fat-saturated T1- and T2-weighted images. Images were processed using AMIDE 2 processing software. Uptake values, presented as percent injected activity per cubic centimeter (%ID/cc) for each organ, were calculated in a 3D region of interest manually drawn using MR images. Imaging was carried out in MC38 and CT26 tumor-bearing mice three days after the last treatment with either PBS or a regimen with immune checkpoint inhibitors (ICI). ICI was given intraperitoneally for three doses at three days apart of anti-PD-1 (250 µg, clone RMP1-14) + anti-CTLA-4 (100 µg, clone 3H3) (Bioxcell, Lebanon, NH, USA).

### 2.4. Therapeutic Studies

A schematic representation of experimental timeline can be found in Figure S1. Mice bearing either MC38 or CT26 subcutaneous tumors were employed for therapy. Groups of 5 female mice were randomized, administered either PBS (control; intravenous), three doses of immune checkpoint inhibitors anti-PD1 (250 μg, clone RPMI-14; Bioxcell) and anti-CTLA-4 (100 μg, clone 9D9; Bioxcell) three days apart (ICI, intraperitoneal), ICI followed by intravenous injection of ^90^Y-GZP at two injected activities, 2.22 MBq (Low Dose) or 22.2 MBq (High Dose), or ICI followed by intravenous injection of free ^90^Y (22.2 MBq). Tumor volume was measured by a blinded researcher with calipers 2–3 times per week. Humane end points were weight loss of more than 20% of initial body weight, tumor growth of more than 500% of its initial size (at start of treatment) and overall health decline. No animals, experimental units or data points were excluded from the analysis.

### 2.5. Toxicity Evaluation

Animal weight was measured twice per week, and general well-being was assessed daily by veterinarian staff. At the end of the study, heart, lungs, kidney, spleen, liver, and tumor were excised from mice of all groups and submitted to the Specialized Histopathology Services of the MGH Pathology Core for processing, sectioning and H&E staining.

### 2.6. Histopathological Analysis of Tumor

#### 2.6.1. Immunohistochemistry

For immunohistochemical studies, tumors were collected in 4% paraformaldehyde for 24 h, then placed in ethanol and embedded in paraffin for sectioning. Hematoxylin and Eosin (H&E), CD3, CD4 and CD8 staining were carried out through standard procedures by the Specialized Histopathology Services of the MGH Pathology Core. Brightfield images at 10× magnification were taken from at least 10 fields of view on a Nikon Eclipse Ti microscope. Images were analyzed on ImageJ software (version 1.8.0_172, Bethesda, MD, USA) to determine staining intensity.

#### 2.6.2. Immunofluorescence

Tumor tissue was also stained against granzyme B after deparaffinization and rehydration. Antigen retrieval was carried out using standard heat-based antigen retrieval techniques [18]. Immunofluorescence staining was carried out according to previously published procedures [15]. After blocking with 5% goat serum, rabbit anti-mouse granzyme B primary antibody (ab255598; Abcam, Cambridge, UK) was incubated overnight at 4 °C. On the following day, washing was carried out followed by incubation with AlexaFluor 647 conjugated goat anti-rabbit IgG secondary antibody (A32733; ThermoFisher, Waltham, MA, USA) at room temperature in a moist dark chamber for 1 h. DAPI (4′, 6-diamidino-2-phenylindole) was used for staining cell nuclei. Semi-quantitative data of immunofluorescence were retrieved by measuring total fluorescence in the red channel (granzyme B) divided by area (integrated density). All images were acquired using Biotek Cytation 5 Cell Imaging Multi-Mode Reader and analyzed through Biotek Gen5 software (Agilent, v3.11, Santa Clara, CA, USA).

#### 2.6.3. Western Blotting

Cells lysates were separated by electrophoresis and transferred to a PVDF membrane. After blocking with 5% BSA, the membrane was incubated overnight with primary antibodies anti-CD8 (ab209775, Abcam), anti-GZB (ab255598, Abcam) and anti-GAPDH (ab9485, Abcam) followed by appropriate secondary antibodies. Antibody detection and quantification were conducted using the iBright™ FL1000 (ThermoFisher) and the iBright analysis software v1.0 (ThermoFisher, Waltham, MA, USA).

### 2.7. Statistical Analysis

A sample size of n = 3 was selected for PET/MR imaging. A minimum sample size of n = 5 was used for the other experiments. Two-tailed student *t*-test was used for statistical analysis. A *p* < 0.05 value was considered for statistical significance. Kaplan–Meier curves and log-rank test were used for overall survival analysis. All statistical analyses were performed using GraphPad Prism 7 (San Diego, CA, USA). Quantitative data are expressed as mean ± standard deviation.

## 3. Results

### 3.1. PET/MR Imaging

**Figure 1:**
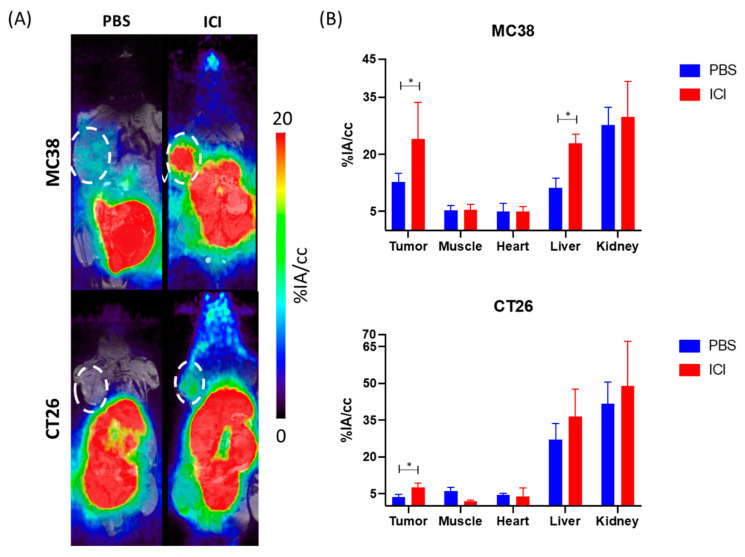
(**A**) Representative PET/MR MIP images of CT26 and MC38 tumor-bearing mice with and without ICI treatment (1 h post injection of 68 Ga-NOTA-GZP). (**B**) Uptake values of blood pool and major organs, showing significantly higher tumor uptake with ICI, represented as %ID/g. * *p* < 0.05.

Longitudinal PET/MR imaging was acquired to access the overall biodistribution of ^68^Ga-GZP and the differential tumor uptake between animals that received PBS and the immune checkpoint inhibitor regimen in two murine models of colon cancer. Figure 1A shows maximum intensity projection (MIP) PET images of the in vivo distribution at 1 h after intravenous injection of the radiotracer in MC38 and CT26 tumor-bearing mice at day 12 after the tumor was implanted. Either PBS or a combination of anti-PD-1 + anti-CTLA-4 was given three times, three days apart (days 3, 6 and 9) and animals were imaged three days after the last treatment. PET imaging revealed a high signal in kidneys and bladder for all groups reflecting its renal excretion as observed previously [13]. Aside from the kidneys, liver was the organ with the highest uptake. A higher tumor uptake in animals that received ICI is observed in both tumor models when compared to the PBS-injected group (Figure S2A). The quantitative region of interest analysis of the PET images (Figure 1B and Figure S2A,B) revealed significantly higher tumor uptake in the ICI-injected group when compared to the PBS group in both MC38 tumor-bearing mice (12.8 ± 2.25 vs. 24.0 ± 9.5%IA/cc, *p* = 0.04) and CT26 tumor-bearing mice (3.7 ± 1.1 vs. 7.5 ± 1.7%IA/cc, *p* = 0.03). Of note, blood pool uptake was not statistically different for any of the groups investigated. Tumor-to-blood ratios were significantly (*p* < 0.05) higher for the group of mice that received immunotherapy when compared to PBS injected only with values of 0.7 ± 0.3 (PBS) vs. 4.1 ± 0.8 (ICI) (*p* = 0.0028) and 2.8 ± 0.7 (PBS) vs. 4.8 ± 0.9 (ICI) (*p* = 0.04) and for CT26 and MC38, respectively (Figure S2C). Tumor-to-muscle ratios (Figure S2D) followed the same trend, reaching 4.1 ± 0.8 and 4.8 ± 2.3 for CT26 and MC38 animals that received ICI, respectively.

### 3.2. Therapeutic Studies

**Figure 2:**
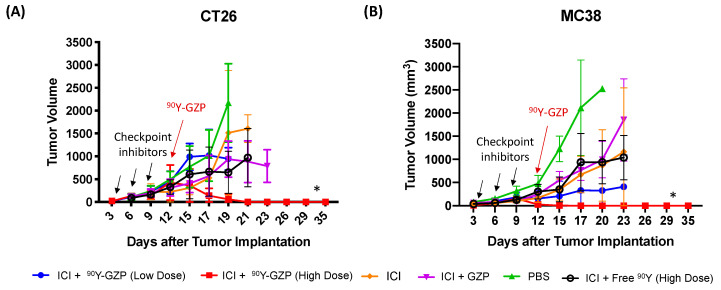
Tumor growth curves of (**A**) CT26 and (**B**) MC38 tumor-bearing mice after administration of the different therapeutic groups. Checkpoint inhibitors were given days 3, 6 and 9 after tumor implantation and ^90^Y-GZP was given on day 12 (indicated by arrows). High dose of ^90^Y-GZP (22.2 MBq) after ICI achieved complete tumor response in both tumor models. * Statistically significant compared to all other groups (*p* < 0.05).

A schematic representation of the therapeutic study groups, dosage and administration regimen can be found in Table S1. For therapeutic studies, animals with MC38 or CT26 tumors were injected with a high (22.2 MBq) and a low (2.2 MBq) dose of ^90^Y-GZP after ICI. The ICI regimen was carried out with the intraperitoneal injection of anti-PD-1 and anti-CTLA-4 (three doses, three days apart, starting three days after tumor implantation). Growth curves are presented in Figure 2. Importantly, both CT26 and MC38 tumor-bearing animals show complete tumor response after treatment with ICI followed by receiving a high dose of ^90^Y-GZP. Animals that received just PBS had exponentially growing tumors throughout the experiment and only survived for around 20 days. In both tumor models, animals that received ICI, ICI + GZP or free ^90^Y had comparable tumor growths that were significantly higher (*p* < 0.05) than the high-dose ^90^Y-GZP group. Of note, a higher dose of ^90^Y-GZP was able to achieve full response and curative treatment in both sets of tumor-bearing mice. In CT26 tumor-bearing mice, a low dose of ^90^Y-GZP was not sufficient to elicit a significant tumor growth delay response and tumors had growth similar to those of the control groups. In contrast, in MC38 tumor-bearing mice, a low dose of ^90^Y-GZP resulted in significantly smaller (*p* < 0.05) tumor volumes when compared to PBS group but not when compared to groups that received ICI.

**Figure 3:**
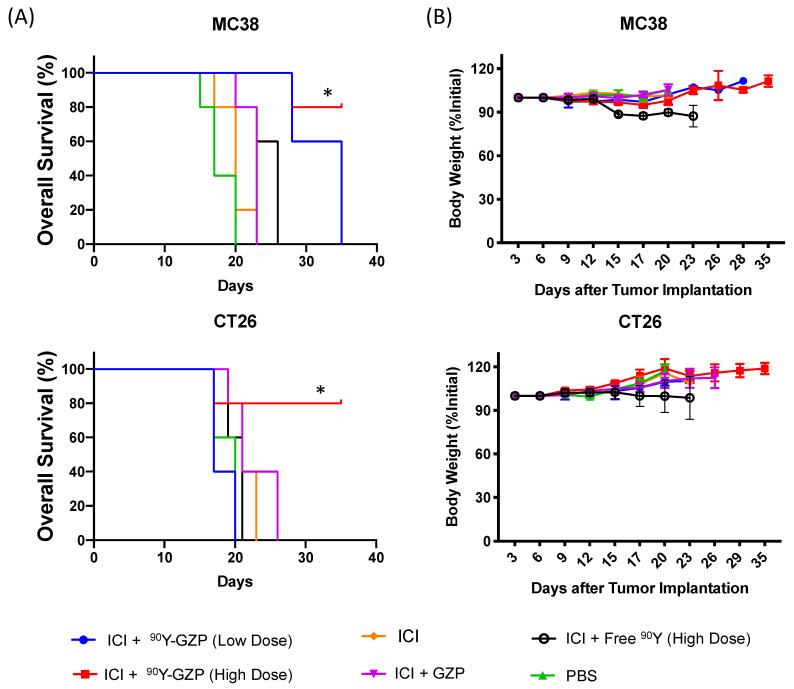
(**A**) Overall survival plots of MC38 and CT26 tumor-bearing mice. Significantly increased overall survival was achieved for ICI followed by 22.2 MBq of ^90^Y-GZP (high dose) in both tumor models as well as ICI followed by 2.2 MBq of ^90^Y-GZP (low dose) in animals bearing MC38 tumors. (**B**) Body weight measurements carried out throughout the study reveal no signs of overall toxicity in any of the treatment groups. * Statistically significant when compared to all other groups at the end of the study (*p* < 0.05).

More importantly, an increased overall survival was found for animals that received a high dose of ^90^Y-GZP in both tumor models investigated (Figure 3A). Median survival remained undefined (>35 days) in CT26 tumor-bearing mice who received a higher dose of ^90^Y-GZP when compared to 20, 21, 21, 21 and 23 days for PBS, ICI + free ^90^Y (high dose), ICI, ICI + GZP and ICI + ^90^Y-GZP (low dose), respectively. Similarly, in MC38 tumor-bearing mice, a median survival was undefined (>35 days) for the ^90^Y-GZP high-dose group, which significantly increased (*p* < 0.05) when compared to 17, 26, 20, 23 and 28 days found for PBS, ICI + free ^90^Y (high dose), ICI and ICI + GZP and ICI groups. In addition, in the MC38 tumor model, animals injected with ICI + ^90^Y-GZP (low dose) also survived longer with a median survival of 28 days when compared to other groups. Since MC38 tumors usually respond well to immunotherapy alone and the tumors were slightly smaller in animals that received ICI + ^90^Y-GZP (high dose) at day 12 when compared to control groups, we performed additional experiments comparing ICI alone and ^90^Y-GZP (high dose) when tumors were a little bigger and statistically identical between those groups (Figure S8). Treatment also resulted in significantly smaller tumor volumes for animals injected with ICI + ^90^Y-GZP (high dose) when compared to the ICI alone group. After tumor tissue collection (Figure S3), an increased necrotic area was observed in tumors of animals injected with a high dose of ^90^Y-GZP when compared to the other groups investigated.

### 3.3. Toxicity Evaluation

Overall systemic toxicity was investigated through differences in body weight. Throughout the experiments, no major changes in body weight were found for any of the groups investigated (Figure 3B) and none of the groups reached the humane endpoint of more than 20% body weight decrease.

**Figure 4:**
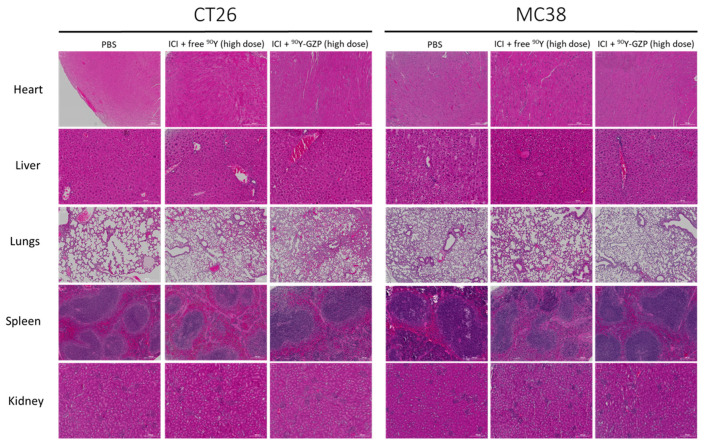
Histological evaluation of major organs reveals no systemic toxicity after both doses of ^90^Y-GZP at either high or low dose. Scale bar = 200 µm.

To further investigate potential toxicities, at the end of the study, major organs were excised and H&E stained. H&E staining of tissue slides of heart, liver, lungs, spleen and kidney (Figure 4) demonstrated no morphological alterations in any of the organs investigated in any of the groups.

### 3.4. Histopathological Analysis of Tumor

**Figure 5:**
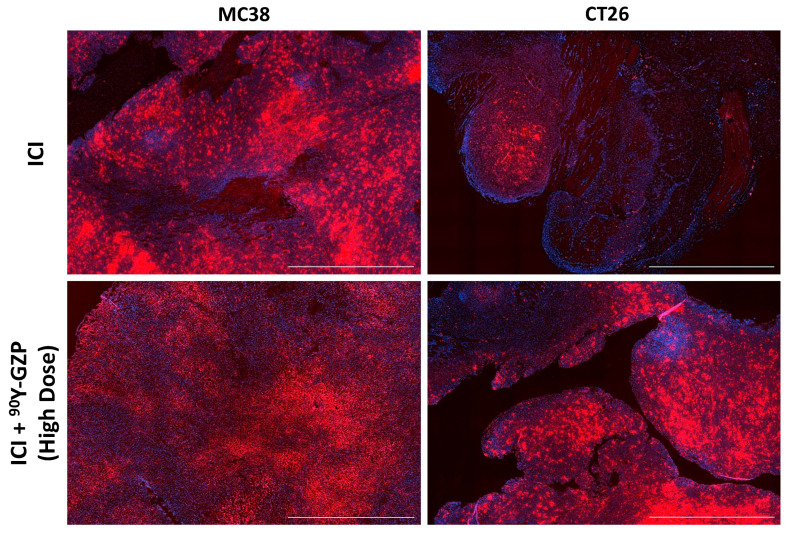
Immunofluorescence staining against granzyme B (red) of tumor samples after ICI therapy only and ICI + ^90^Y-GZP (high dose) demonstrating high levels of granzyme B presence within the tissue. Nuclei are stained with DAPI (blue). Scale bar = 200 µM.

CD3, CD4 and CD8 IHC staining was performed in tumor tissues collected at different time points after treatment (Figure S1). Representative images of CD3, CD4 and CD8 staining of tumor tissues in animals that received ICI, ICI + lose-dose ^90^Y-GZP and high-dose ^90^Y-GZP can be seen in Figures S4 and S5. Heterogeneous staining was found within the groups, indicating that some animals responded to ICI therapy and some animals did not. In the same manner, a low or high dose of ^90^Y-GZP did not deplete the amount of immune cell infiltrate within the tumors and in those animals that responded to ICI, ^90^Y-GZP increased expression of CD4 and CD8. Similar levels of these markers were found in all three groups, indicating that targeted radionuclide therapy with ^90^Y-GZP did not deplete immune cell levels within the tumors on the days analyzed. Relatively high levels of CD4 and CD8 were found in animals that received a high dose of ^90^Y-GZP compared to the groups that received a lower dose of ^90^Y-GZP. Immunofluorescence staining of tumor tissue (Figure 5 and Figure S7) also demonstrated that very high levels of GZB were present in animals that received ICI, indicating good response to immunotherapy and good potential for our targeting agent to deliver a toxic radiation dose to the tissue, as well as high levels of GZB present after targeted radiotherapy with ^90^Y-GZP. These results indicate that CD8 T cells remain active or are additionally recruited after targeted radionuclide therapy. Western blot studies were carried out to validate findings from IHC and the results (Figure S6) demonstrate that, in MC38 tumor-bearing mice, no statistical significance was found for CD8 and GZB levels of animals that received ^90^Y-GZP when compared to animals that received ICI only. In contrast, in CT26 tumor-bearing mice, even though levels of CD8 were not significant between groups, slightly lower levels of GZB were found for the animals that received ^90^Y-GZP after ICI.

## 4. Discussion

Metastatic colorectal cancer (mCRC) often presents with multiple metastatic sites that are unresectable [19]. In colon cancer, pembrolizumab and nivolumab (anti-PD1 antibodies) have been FDA approved as monotherapy or combined with ipilimumab (anti-CTLA-4 antibody) as treatment for patients with microsatellite instability high (MSI-H)/DNA mismatch repair-deficient (dMMR) mCRC. Unfortunately, ICI’s clinical benefit is somewhat limited in mCRC as most (>95%) mCRC patients are microsatellite stable (MSS)/DNA mismatch repair-proficient (pMMR) (MSS/pMMR). Several mechanisms of resistance to ICI therapies have been proposed in CRC, but the factors that drive MSS/pMMR patients to be resistant to ICI are still unknown [20]. In addition, even among MSI/dMMR patients, heterogeneity in response to ICI has been demonstrated, either through a primary resistance mechanism or because of a misdiagnosis of their MSI or dMMR status. Therefore, to expand and improve the clinical outcomes of mCRC patients that receive ICI, we need to either (i) develop a better method of identifying patients that will most likely respond to ICI or (ii) combine ICI with other forms of therapy for a synergistic therapeutic response.

Chemotherapy and radiotherapy (RT) are known to damage tumor cells, which can lead to dendritic cells recognition of tumor antigens and CD8+ T cells activation [21,22]. Hence, combining ICIs with chemotherapy or RT could potentially be synergistic and overcome ICI resistance in patients with mCRC. RT for mCRC is mostly performed through an external beam irradiation (EBRT—External Beam Radiation Therapy), which is limited in the number and size of tumor lesions it can treat, as treatments with large radiation fields are associated with significant toxicity [23]. Unlike EBRT, which often cannot target the entire metastatic burden, ligand-directed molecularly targeted radionuclide therapy (TRT) is a radiation therapy modality consisting of systemic delivery of radioactive atoms to induce DNA damage in tumor cells. Consequently, TRT can deliver its tumoricidal radiation irrespective of tumor location and burden in a selective manner that minimizes normal tissue damage. TRT agents typically target specific receptors and metabolic transporters overexpressed in tumor cells [24]. Therefore, this systemic mode of radiation delivery is optimally suited for targeting metastatic lesions. Several targets have been proposed for mCRC [25], but its success is hampered by lack of specificity, sensitivity, and tumor heterogeneity [26,27,28]. We overcome the limitation that many mCRC tumor cells lack specific targets addressable by TRT by utilizing a target that will potentially be present after ICI in the tumor microenvironment. Our group has previously demonstrated that an anti-granzyme B peptide (GZP) specifically binds to granzyme B, a marker of CD8+ T cell activation. ICI ultimately works by promoting activation of CD8+ T cells to produce and release granzyme B resulting in tumor cell toxicity.

Herein, we aimed at using radiolabeled GZP as a novel theranostic agent for CRC in combination with ICI therapy. Our results reveal that ^68^Ga-GZP successfully accumulated in two tumor models of CRC after ICI. We further show that there was a statistically significant higher tumor uptake in animals after ICI treatment when compared to animals without ICI treatment. We also observed intrinsic differences between CT26 and MC38 tumor uptake, with the latter having a significantly higher uptake. These data are in agreement with literature findings that MC38 tumors are usually more immunogenic and respond better to ICI [29]. We hypothesized that activated T cells could not only be used for patient selection, but could also be harnessed for selective targeted radiotherapy. For therapeutic purposes, tissue depth penetration and tumor dosimetry are favorable for β− emitters, with isotopes such as ^90^Y extensively used in clinical settings [12]. Since we confirmed higher tumor uptake of GZP after ICI therapy, we administered two different doses of ^90^Y-GZP after ICI to investigate if TRT could contribute to antitumor effects and have a synergistic relation with ICI. Indeed, we found that in both tumor models, a high dose of ^90^Y-GZP promoted total tumor regression and increased survival, a major potential advantage when treating such tumors. Interestingly, a low dose of ^90^Y-GZP contributed to a slightly (not statistically significant) worse antitumor response than ICI alone in CT26 tumors. Radiotherapy can promote both stimulatory and immunosuppressive response in immune cells [30,31]. Dose, fractionation and tumor type likely influence pro- or anti-inflammatory radiotherapy traits [32,33,34]. However, a low dose of ^90^Y-GZP actually contributed positively to tumor regression in MC38 tumors, most likely because MC38 is more immunogenic and had significantly higher tumor uptake so that MC38 tumors likely received a much higher dose of radiation than CT26 tumors, even if the same activity was injected for both. A dosimetry study is indicated to identify the actual dose received by each tumor model. Notably, this is one of the biggest advantages of a theranostic approach—by screening tumor uptake before TRT, it is possible to calculate in a personalized manner how much of radioactivity should be injected to achieve a dose capable of tumor eradication, especially when synergistic with ICI. It has also been shown that the type of dosage (fractionated or single dose) as well as timing between radiotherapy administration and ICI can influence antitumor response since it influences lymphoid and myeloid responses and CD8+/T reg ratios along with modulation of PD-L1 and other suppression/activation markers. In future studies, we plan to better understand the effects of dose timing and fractionation to optimize outcomes. We will also use metastatic models to see if this approach can also treat multiple tumor sites and diminish overall metastatic burden. We also understand that ^68^Ga may not be the perfect surrogate for ^90^Y. However, since imaging with ^90^Y is not feasible and ^68^Ga is a widely available PET isotope, ^68^Ga is routinely used as a theranostic “twin” to therapeutic isotopes such as ^177^Lu and ^90^Y in both preclinical and clinical settings [35,36,37,38]. For example, a NETSPOT (^68^Ga-DOTATATE) scan is routinely used in the clinic as a theranostic “twin” in the identification of neuroendocrine patients that will benefit from treatment with Lutathera (^177^Lu-DOTATATE) [39], and Locametz or Illuccix (^68^Ga-PSMA-11) are also FDA-approved complementary diagnostic imaging agents for radioligand therapy with Pluvicto (^177^Lu-PSMA-617) [40] in metastatic castration-resistant prostate cancer. Several studies and clinical trials also use ^68^Ga-PSMA agents as companion diagnostics for PSMA-based radionuclide therapy ^225^Ac (clinical trials NCT04597411 and NCT04886986). Further studies with ^86^Y-GZP are potentially needed for confirmation of similar pharmacokinetics and dosimetry calculations of ^90^Y-GZP.

We evaluated the toxicity of our combinational treatment through morphological changes to major organs and found no signs of toxicity in any of the groups or organs, including the liver which had the highest off-target radioprobe uptake. However, one CT26 tumor-bearing mouse and one MC38 tumor-bearing mouse administered with ICI + ^90^Y-GZP high dose died of unknown causes during our study. Other animals injected with ^90^Y-GZP (low dose) also reached humane endpoints due to overall health decline and had to be euthanized. Further toxicity studies such as complete blood count and comprehensive metabolic panel are needed to elucidate potential toxicities.

It has been demonstrated in preclinical settings that radiation can also have immunosuppressive effects and a few studies have found diminished response to ICI after radiotherapy [41]. Even though a thorough investigation of the changes of the tumor immune microenvironment after TRT + ICI is out of the scope of this study, we stained tumor tissues of different treatment groups for basic immune cell phenotypes. We demonstrate that the presence of immune cell infiltrates was not depleted (or was replenished to a level above that at pre-radiotherapy treatment) after TRT. We also noted that high granzyme B levels were still present in tumor tissues after ^90^Y-GZP as measured by immunofluorescence in both tumor models, but lower levels of granzyme B were found in CT26 tumor-bearing mice as measured by Western blot (WB). It is known that activated T cells transiently release GZB into a pericellular space that can be internalized by a target cell or can be inhibited or diffuse from the tumor [42,43]. The active granzyme B in the extracellular tumor microenvironment is dynamic, and variation in GZB levels might be due to timing rather than downregulation of GZB expression. Further studies are needed to understand the immunomodulatory effects of TRT in combination with ICI at different time points and with different administration regimens (fractionated doses, for example).

## 5. Conclusions

PET/MR imaging allowed clear visualization of activated CD8+ T cells by imaging granzyme B expression with ^68^Ga-GZP after immune checkpoint inhibitor therapy. In our animal models of colorectal cancer, ICI was not sufficient to promote complete response in two different tumor models. However, when ICI was combined with a relatively high dose of targeted radionuclide therapy (^90^Y-GZP), animals in both tumor models (MC38 and CT26) had complete and durable tumor regression and increased overall survival. Treatments were also well tolerated as no morphological changes were found in major organs of the animals. In summary, this study demonstrates that GZP can be used as a theranostic agent. When labeled with ^68^Ga, it can select responders to immune checkpoint inhibitors and, when labeled with ^90^Y, it can improve the therapeutic efficacy of ICI.

## Data Availability

Not applicable.

## Supplementary Materials

The following supporting information can be downloaded at: https://www.mdpi.com/article/10.3390/pharmaceutics14071460/s1, Figure S1: Schematic representation of studies workflow; Figure S2: PET/MR inserts, tumor-to-blood and tumor-to-muscle ratios. Figure S3: H&E staining of MC38 and CT26 tumor tissues. Figure S4: IHC staining of CD3, CD4 and CD8 on CT26 tumor tissue. Figure S5: IHC staining of CD3, CD4 and CD8 on MC38 tumor tissue. Figure S6: Western Blot quantitative data. Figure S7: Semi-quantitative analysis of immunofluorescence staining of granzyme B in tumor samples. Figure S8: Tumor growth curves of MC38 tumor-bearing mice. Table S1: Groups and Doses for the Therapeutic Sudy.
