# Supplementary material for: Immune Checkpoint Inhibitor-Mediated Cancer Theranostics with Radiolabeled Anti-Granzyme B Peptide
Source: Pharmaceutics. 2022 Jul 13;14(7):1460. doi: 10.3390/pharmaceutics14071460 (PMC9325142; doi:10.3390/pharmaceutics14071460)
Supplement: Supplementary file 1 [file pharmaceutics-14-01460-s001.zip › pharmaceutics-1776904-supplementary.pdf]

Immune Checkpoint Inhibitor–mediated Cancer Theranostics with radiolabeled anti-Granzyme B Peptide

Carolina A. Ferreira[1], Pedram Heidari[1], Bahar Ataeinia[1], Nicoleta Sinevici[1], Alyssa Granito[1], Hritik Mahajan Kumar[1], Eric Wehrenberg-Klee[1], Umar Mahmood[1*]

[1] Department of Radiology, Massachusetts General Hospital, Boston, Massachusetts
*corresponding author: Umar Mahmood, M.D., Ph.D. Department of Radiology Massachusetts General Hospital Boston, MA Tel: 617-726-6477 Email: umahmood@mgh.harvard.edu

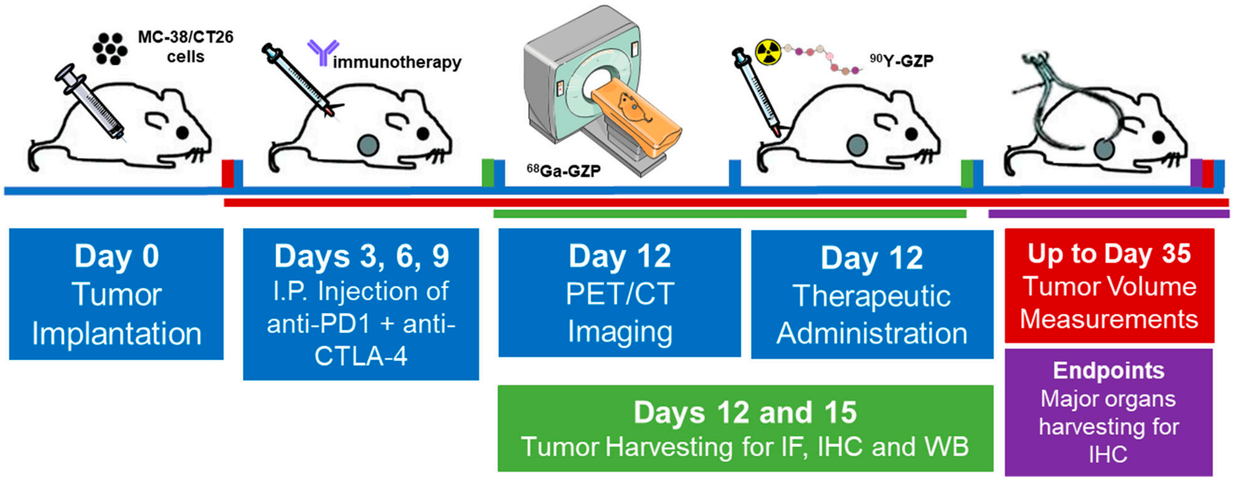

Figure S1. Schematic representation of studies workflow. MC38 and CT26 xenografts were implanted at Day 0. ICI injections were carried out on days 3, 6 and 9. ^{68}Ga-GZP and PET imaging or ^{90}Y-GZP for therapy studies were administered on Day 12. Tumor harvesting for IF, IHC and WB was carried out 3 days after last treatment (days 12 or 15).

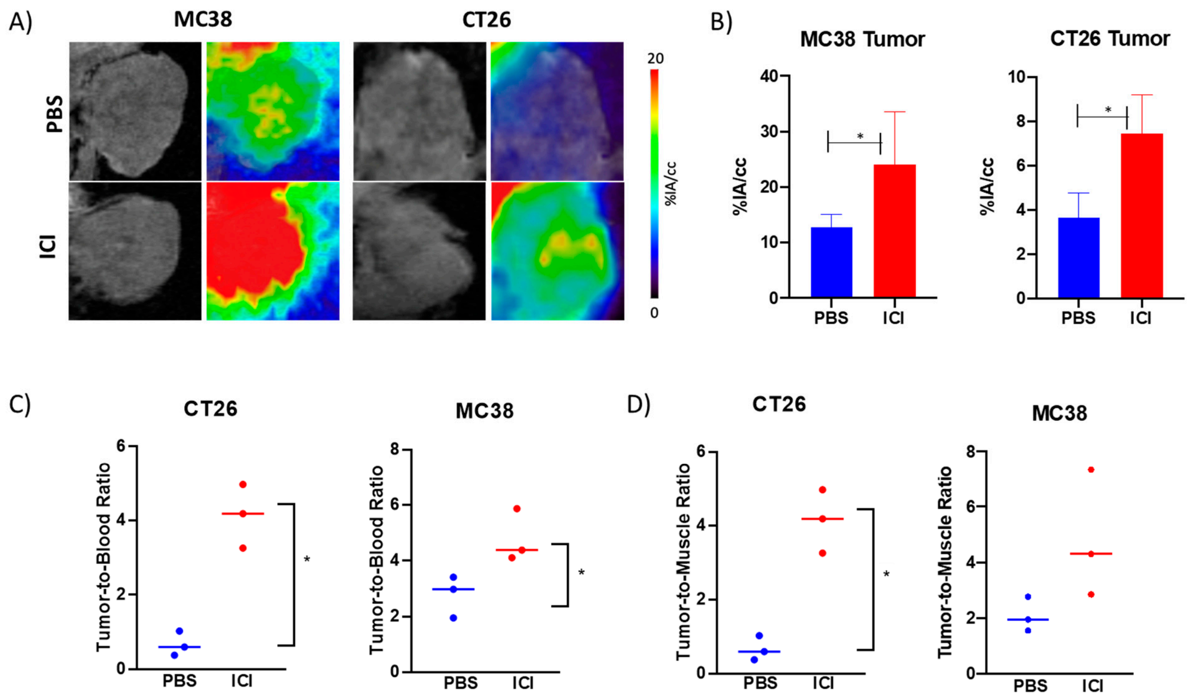

Figure S2. A) PET/MR insert of tumor region and B) ROI values of tumor uptake. C) Tumor-to-blood and D) Tumor-to-Muscle ratios for both tumor models. * $p < 0.05$

Table S1. Groups and Doses for the Therapeutic Study

Group	Treatment	Administration	Day of administration
PBS	PBS (100µL)	i.v.	12
ICI	Anti-PD1 (250µg)<br>Anti-CTLA-4 (100µg)	i.p.	3, 6 and 9
ICI + GZP	Anti-PD1 (250µg)<br>Anti-CTLA-4 (100µg)<br>GZP	i.p. (ICI)<br>i.v. (GZP)	3, 6 and 9 (ICI)<br>12 (GZP)
ICI + ^{90}Y-GZP<br>(Low Dose)	Anti-PD1 (250µg)<br>Anti-CTLA-4 (100µg)<br>^{90}Y-GZP (2.2MBq)	i.p. (ICI)<br>i.v. (^{90}Y-GZP)	3, 6 and 9 (ICI)<br>12 (^{90}Y-GZP)
ICI + ^{90}Y-GZP<br>(High Dose)	Anti-PD1 (250µg)<br>Anti-CTLA-4 (100µg)<br>^{90}Y-GZP (22.2MBq)	i.p. (ICI)<br>i.v. (^{90}Y-GZP)	3, 6 and 9 (ICI)<br>12 (^{90}Y-GZP)
ICI + ^{90}Y<br>(High Dose)	Anti-PD1 (250µg)<br>Anti-CTLA-4 (100µg)<br>^{90}Y (22.2MBq)	i.p. (ICI)<br>i.v. (^{90}Y)	3, 6 and 9 (ICI)<br>12 (^{90}Y)

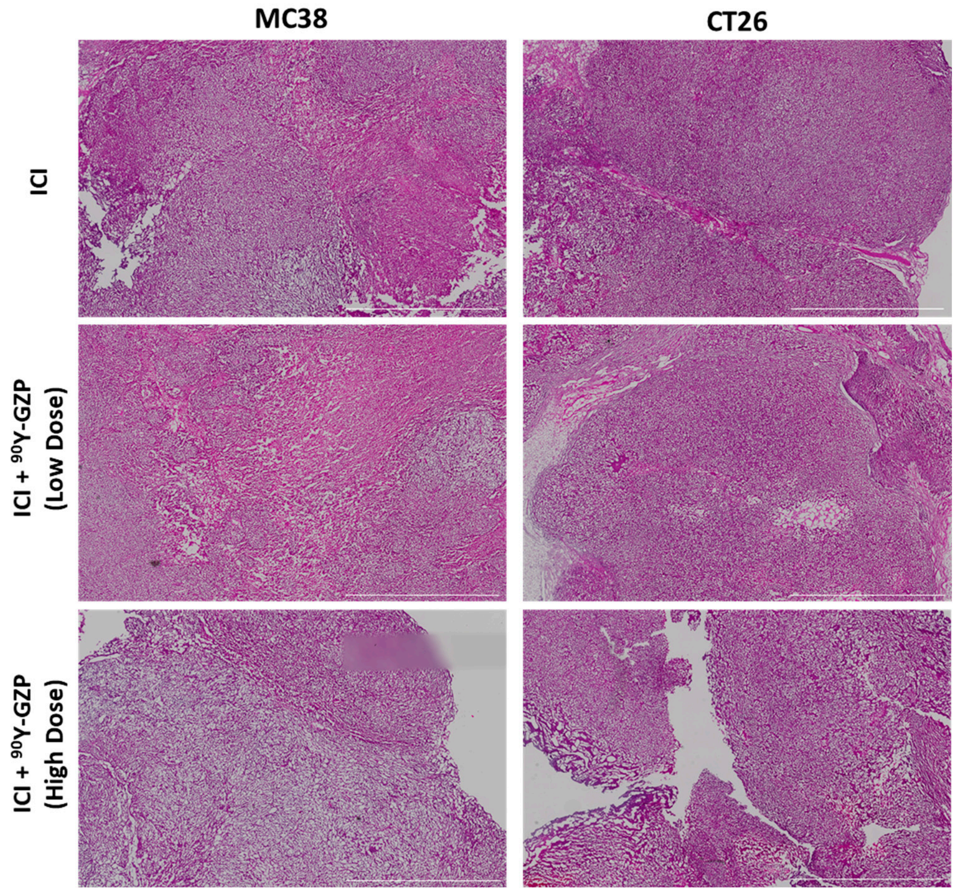

Figure S3. H&E staining of MC38 and CT26 tumor tissue, collected at day 15 demonstrating increased necrotic areas in animals injected with ICI + ^{90}Y-GZP (high dose).

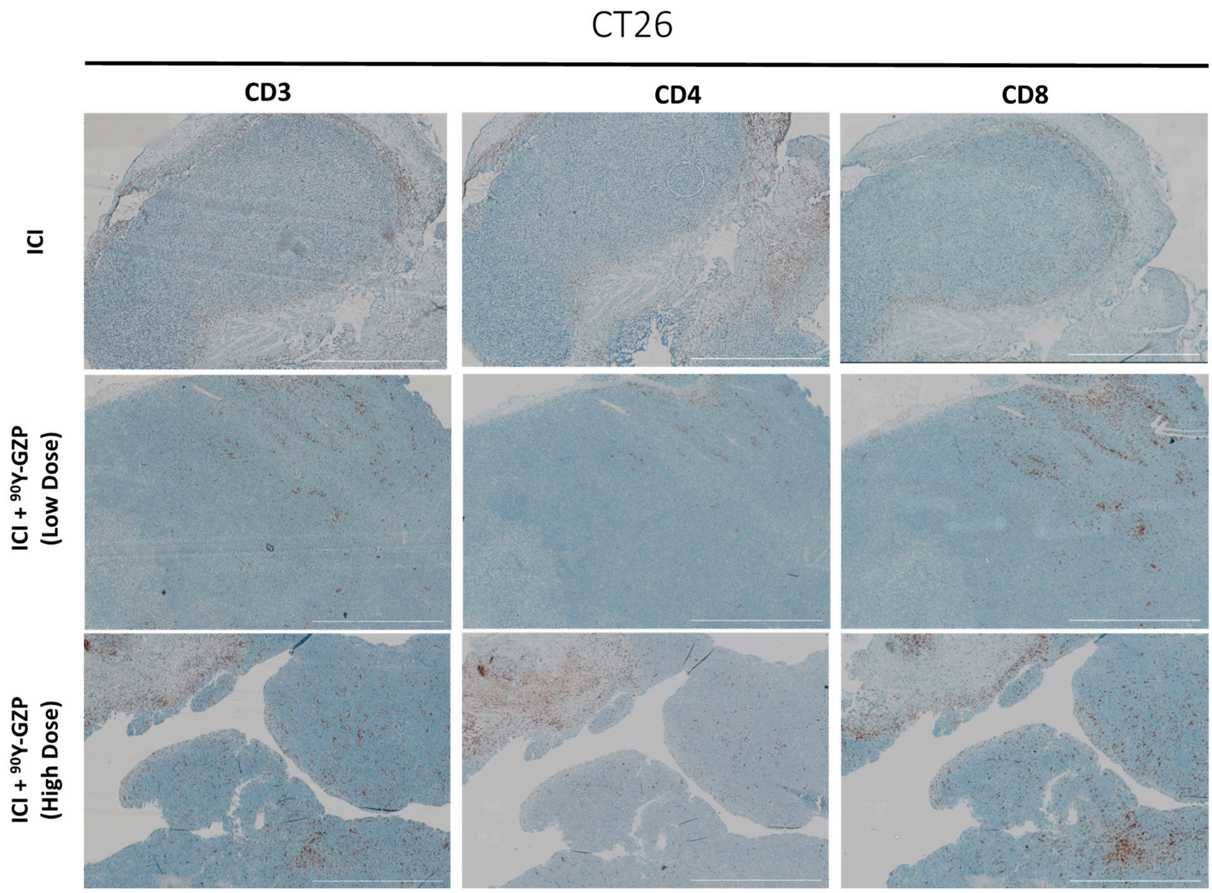

Figure S4. Immunohistochemistry staining of CD3, CD4 and CD8 on CT26 tumor tissue collected 3 days after therapeutic administration (day 12 for ICI and day 15 for ICI + ^{90}Y-GZP).

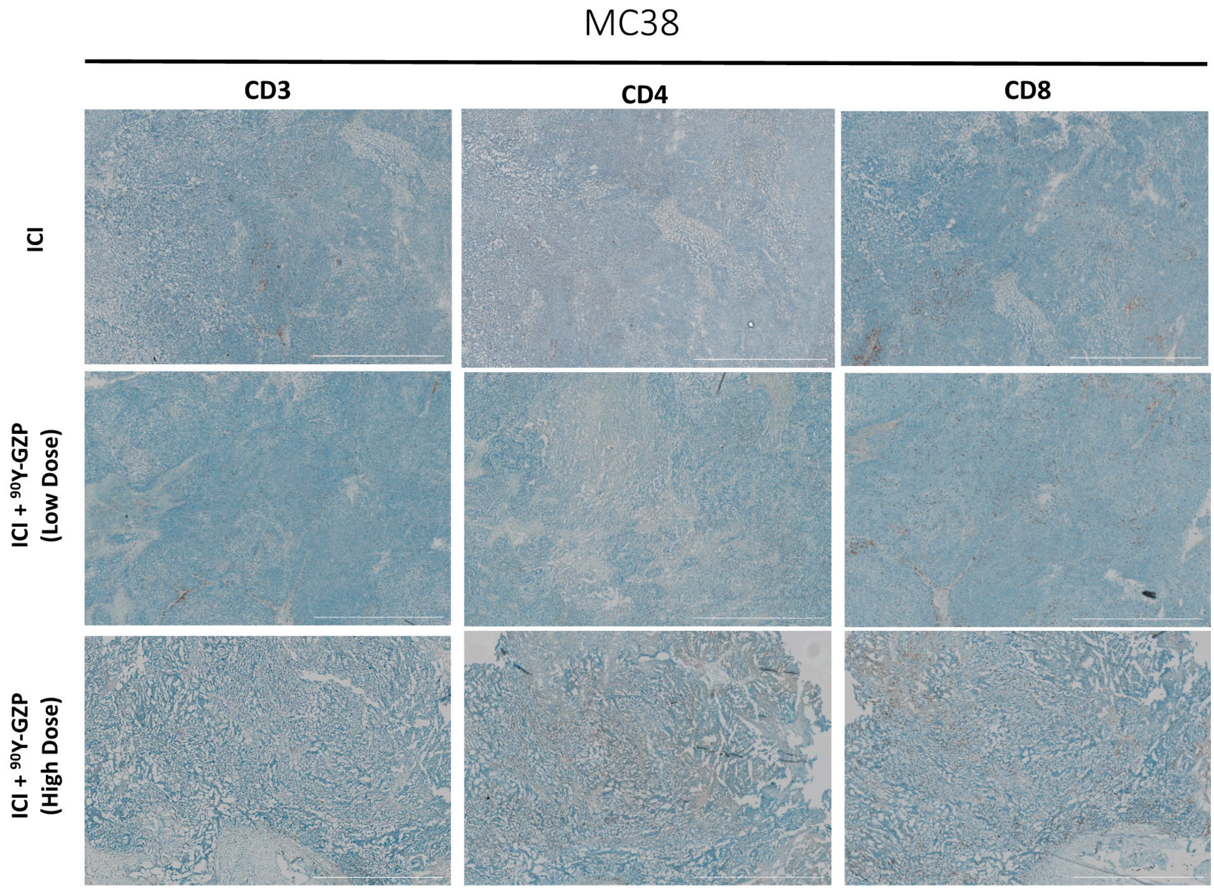

Figure S5. Immunohistochemistry staining of CD3, CD4 and CD8 on MC38 tumor tissue collected 3 days after therapeutic administration (day 12 for ICI and day 15 for ICI + ^{90}Y-GZP).

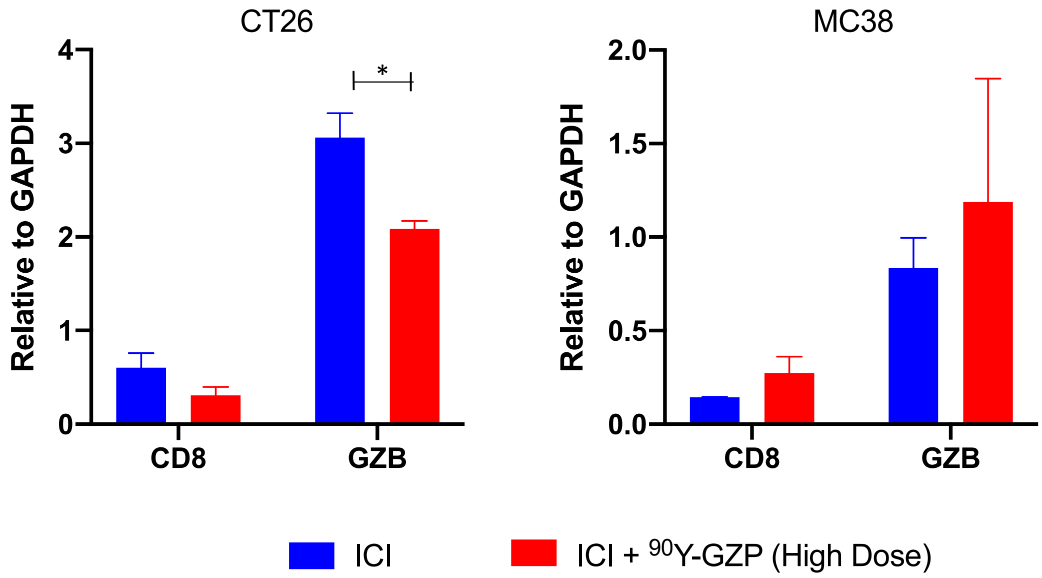

Figure S6. Western Blot quantitative data depicting CD8 and GZB expression in CT26 and MC38 tumor bearing mice that received either ICI or ICI + ^{90}Y-GZP (High dose). *$p < 0.05$

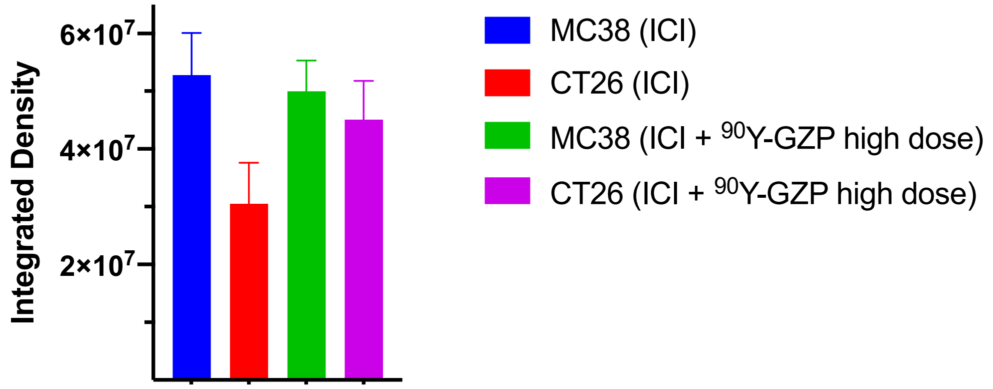

Figure S7. Semi-quantitative analysis of immunofluorescence staining of granzyme B of tumor samples of CT26 and MC38 tumor bearing mice after ICI and ICI + ^{90}Y-GZP high dose.

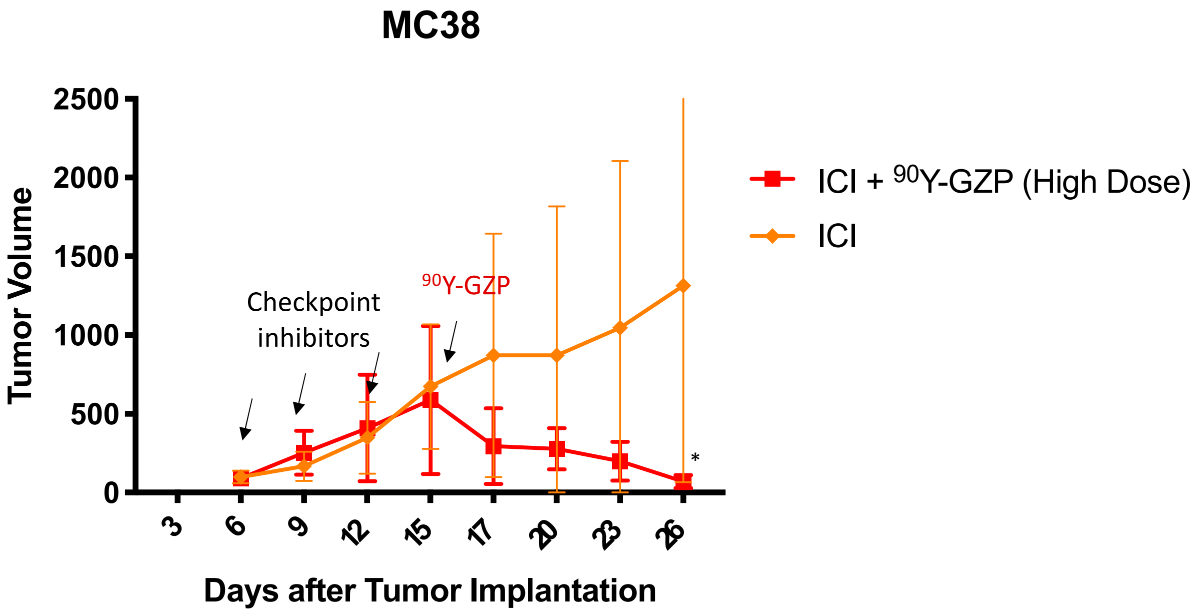

Figure S8. Tumor Growth Curves of MC38 tumor bearing mice after administration of the different therapeutic groups. Checkpoint inhibitors were given days 6, 9 and 12 after tumor implantation and ^{90}Y-GZP was given on day 15 (indicated by arrows). *Statistically significant than all the other groups ($p<0.05$).
